# Managing the combined consequences of COVID-19 infection and lock-down policies on athletes: narrative review and guidelines proposal for a safe return to sport

**DOI:** 10.1136/bmjsem-2020-000849

**Published:** 2020-10-19

**Authors:** Jean-Bernard Fabre, Laurent Grelot, William Vanbiervielt, Julien Mazerie, Raphael Manca, Vincent Martin

**Affiliations:** 1 Human Motion Analysis, Humanfab, Aix-en-Provence,France; 2 Health and Safety Department, Aix-Marseille-University,Marseille, France; 3 Physical Therapy, Humanfab, Aix-en-Provence,France; 4 Institut Universitaire de France (IUF), Paris, France; 5 Université Clermont Auvergne, AME2P, Clermont-Ferrand,France

**Keywords:** Physiology, cardiology, exercise, respiratory, sports & exercise medicine

## Abstract

COVID-19 pandemic is a global health matter. The disease spread rapidly across the globe and brought the world of sports to an unprecedented stoppage. Usual symptoms of the disease are fever, cough, myalgia, fatigue, slight dyspnoea, sore throat and headache. In more severe cases, dyspnoea, hypoxaemia, respiratory failure, shock and multiorgan failure occur. This appears to be a self-limiting phenomenon related to individuals with coexisting medical conditions, such as hypertension, diabetes and cardiovascular disorders. Nevertheless, cases have been reported in professional soccer players in extremely good fitness condition, demonstrating that athletes are not spared by the disease. Despite COVID-19 clinical manifestations are mainly respiratory, major cardiac complications are being reported, leading to acute myocarditis. One difficulty is that symptoms of COVID-19 vary among individuals, with athletes being affected with no apparent sign of the disease. This could be a real danger for amateur or professional athletes when returning to their usual training and thus to play. Another threat is that the lock-down policies did not allow most athletes to follow their usual training routines. There is thus a need for a careful approach by the sports medicine community to ensure safety of all athletes before they return to sport. Here, we propose evaluation guidelines of fitness and health of athletes to (1) reduce any lethal risk of practice, especially myocarditis and sudden cardiac death; (2) evaluate the combined consequences of the disease and detraining on the physical abilities and biological profile of athletes; and (3) monitor postinfection fatigue symptoms.

## INTRODUCTION

The SARS-CoV-2 (COVID-19) pandemic is a global public health emergency. According to the data available, from the beginning of the epidemic to 23 August 2020, a total of 23  256  567 people were infected, and 805 733 died in 202 countries, as reported by the Coronavirus Resource Centre of the John Hopkins University of Medicine.^1^ Many research teams proposed that COVID-19 and β-coronaviruses are from the same family^2 3^ with a 79.0% nucleotide identity to SARS-CoV and 51.8% identity to MERS–coronavirus (MERS).^4^ Moreover, research have demonstrated that COVID-19 genome has 96% of similarity with the entire genome of bat coronavirus.^5^ The SARS-CoV-2 is predominantly spreading through the respiratory tract and uses the same receptor as SARS-CoV (ACE2). The principal source of contagion is most likely human-to-human aerosol transmission, which happens essentially through contaminated droplets, hands or surfaces. Virus particles present in secretions from an infected person’s respiratory system infect others through direct contact with mucous membranes.^6^ Also, the median incubation period is between 2 days and 12 days (median 5.1 days). Thus, based on the data from the previous coronavirus epidemics (SARS-CoV and MERS), COVID-19 has first been described as influenza viral infections and a pathogen that primarily targets the respiratory system (ie, type 2 pneumocytes) with pneumonia and acute respiratory distress syndrome (ARDS) for the most severe cases.^7^ After few months and a better understanding of the disease, it appears that COVID-19 also causes direct myocardial injury,^8^ disseminated intravascular coagulation and multiorgan failure.^9^ One of the main difficulties is that symptoms of COVID-19 vary among individuals, and even young athletes can be infected with no apparent symptom of the disease. This could be a real danger for amateur or professional athletes when returning to their usual training and subsequently when they will return to intensive play. Indeed, as an emerging infectious disease, COVID-19 late effects and sequelae are still unknown for both the moderate and severe forms. The disease spread rapidly across the globe owing to the unique properties of the virus (ie, extraordinary genetic diversity, highly contagious, easy spreading, relative resistance to climatic variations),^10^ causing an unprecedented pandemic forcing governments to impose an almost global quarantine. At the beginning of 2020 (January–March), the whole world, including the world of sports, entered an extreme and unknown situation, where, gradually, all sports competitions were postponed and any organised training or practice was banned.^11^ In the absence of a vaccination or an antiviral therapy, physical distancing emerged as the key step against COVID-19. Isolation, of course, did not allow athletes to follow their usual training and competitive routines. Regardless of duration, isolation could have a significant impact on the physical and mental state of an athlete. Staying in quarantine can have negative effects on physiological systems, especially aerobic capacities, muscular performance and body composition. This well-known phenomenon is called detraining.^12^ After at least 4 weeks of quarantine, depending on country lock-down measures, the rates of new infection begin to plateau and even decline in some countries. This glimmer of hope has created an enthusiasm and a collective euphoria about the resumption of normal sports activity. To reduce injury risk and promote the return of performance, having a better understanding of the combined effect of detraining and COVID-19 infection seems to be relevant. Hence, there is an emergency for a global and purposeful approach to help the sports medicine community to ensure safety and wellness of all athletes before they return to sport. We propose here a global model of evaluation to (1) reduce any lethal risk of practice, especially myocarditis and sudden cardiac death (SCD); (2) evaluate the combined consequences of the disease and detraining on the physical abilities and biological profile of athletes; and (3) monitor postinfection fatigue symptoms.

## COVID-19 SYMPTOMS AND COMPLICATIONS

Usual symptoms of the disease are fever, cough, myalgia, fatigue, slight dyspnoea, sore throat, headache and conjunctivitis.^13–15^ Gastrointestinal involvement was also reported in a smaller percentage of cases, with diarrhoea, nausea and vomiting.^16^ Only around 10% of cases become serious, with dyspnoea, hypoxaemia and an extensive (>50%) destruction of the lung parenchyma leading to fibrosis.^17^ In around 5% of cases, the disease evolved in a critical condition, with respiratory failure, pneumonia, shock, multiorgan failure and, in the most serious cases, death, which is almost always caused by progression to ARDS and multiorgan failure.^18–20^ Acute hypoxaemia may cause obstinate dyspnoea with the need for oxygen therapy administration through high-flow nasal oxygen, or through the application of a non-invasive positive pressure (with oronasal or face masks, helmets).^21 22^ Unfortunately, in the case of O_2_ saturation worsening, orotracheal intubation and invasive mechanical ventilation are mandatory in intensive care unit. However, SARS-CoV-2 infection does not only affect lungs.

## CARDIOVASCULAR COMPLICATION OF COVID-19

Despite COVID-19 clinical manifestations are essentially respiratory, major cardiac and vascular (ie, vasculitis) complications are being reported. One of the proposed mechanisms of myocardial injury includes a cytokine storm that develops during severe COVID-19 illness. This may lead to decrements in cardiac function, similar to those seen in other forms of sepsis, with features that overlap with classic forms of ‘stress’ or catecholamine-induced cardiomyopathy.^23^ To date, this appears to be a self-limiting phenomenon confined to the severe phases of the illness in individuals with coexisting medical conditions, such as hypertension, type 2 diabetes and cardiovascular disorders.^15 24^ Alternatively, COVID-19 may directly infect myocardial cells, thereby leading to myocarditis with lymphocyte-rich inflammatory histology, acute impairment of cardiac muscle function and potentially residual chronic scars with increased vulnerability to malignant ventricular arrhythmias. Importantly, myocarditis has recently been reported in patients who did not present symptoms, such as fever or respiratory difficulties.^25^ One of the major difficulties is that symptoms of COVID-19 vary among individuals.^18^ Lavezzo and colleagues (unpublished data, 2020) reported in a cohort study conducted in the town of Vò Euganeo in Italy that around 50–75% of individuals with positive RT-PCR throat swab results remain asymptomatic, while others develop mild influenza-like symptoms. It is therefore difficult to differentiate COVID-19 from other respiratory diseases,^26 27^ which may be a real threat. Interestingly, many professional team sport players in extremely good fitness condition have been infected (unpublished data, French Soccer Federation, 2020), demonstrating that athletes are not spared by the disease. Although definite evidence is lacking, athletes may beat higher risk of developing myocarditis than the general population. Sports activity can influence the susceptibility to infections, depending on the intensity and duration of the physical exercise.^28^ While moderate physical activity may improve immunological defences,^28 29^ intense and prolonged training or competition lower the immunity by reducing salivary secretory IgA, lactoferrin and lysozyme altering the T-cell response.^29^ All mechanisms may increase the vulnerability of athletes to viral infections.^29 30^ The myocarditis associated with COVID-19 may be an additional threat since acute cardiac injury is observed in almost one-fifth of patients, with a 50% survival rate.^31^ Myocarditis has been traditionally considered as the cause of life-threatening ventricular arrhythmias and SCD in athletes. The prevalence and acute and delayed clinical implications among infected people who experience mild illness or who remain asymptomatic remain completely unknown. Also unknown is the incidence of silent myocardial inflammation that lingers long after the resolution of typical COVID-19 symptoms, a form of disease that may uniquely affect athletes during resumption of training and competition.

This could be a real danger for amateur or professional athletes. There is a need to prevent lethal risk, especially myocarditis and SCD risk, prior to any practice.

## CARDIAC TESTING IN ATHLETES PREVIOUSLY INFECTED WITH COVID-19

From the perspective of heart health, the preparticipation evaluation (PPE) is traditionally considered as a tool to screen for occult cardiovascular diseases that predispose the athlete to SCD.^32^ In the context of COVID-19, it would be prudent to adopt a broader approach of the cardiovascular PPE. In addition to using the PPE to search for rare genetic and congenital conditions, it is recommended to look for cardiovascular sequelae of COVID-19 to ensure a safe return to sport. Though imperfect, medical history and physical examination are valuable tools for identifying athletes with underlying myocardial inflammation and/or overt myocarditis. The use of 12-lead ECG is crucial in the initial evaluation of athletes. Specialist must seek patterns that reflect myocardial inflammation such as T-wave inversions and new ST segment changes.^33^ Repolarisation abnormalities are the most common alterations in acute myocarditis, being detected in 40% of the patients. The most notable ECG feature of early repolarisation is ST segment elevation, which may vary in terms of morphology, location and degree. T-wave inversion can also be observed particularly in black athletes but confined to leads V1–V4.^34^ For more details about acute myocarditis and ECG evaluation, readers should refer to the excellent review of Vio and colleagues.^35^

However, as failure to identify athletes with myocarditis by preparticipation screening ECG has been reported before,^36^ blood sampling as a complementary approach during the PPE is recommended.^37 38^ Biomarkers indicative of myocardial injury are indeed elevated in myocarditis. In a recent meta-analysis review about the effects of COVID-19 on cardiac biomarkers, Li and colleagues^16^ found troponin I significantly increased in patients with COVID-19 with severe disease compared with those with milder infection. Increases in troponin I, creatine kinase (CK-MB) and N-terminal pro b-type natriuretic peptide (NT-pro BNP) are indicators of possible cardiac damage during COVID-19 infection, and three case reports have found fulminant myocarditis^39 40^ and cardiac tamponade^41^ after COVID-19 infection. We then suggest that troponin I, CK-MB and NT-pro BNP must be routinely measured in infected athletes before they return to play.

This will help to identify those infected athletes that may require additional testing and medical care prior to return to play. However, allowing athletes to return to practice on the sole basis of cardiological assessment may underestimate risks since the COVID-19 can lead to multiorgan failure. There is thus a need for a broader monitoring prior to return to practice.

## MULTIORGAN THREAT AND GLOBAL ASSESSMENT FOR INFECTED ATHLETES

In about 5% of the cases, SARS-CoV-2 infection does not only affect lungs and heart. As commonly observed in sepsis, liver and kidney alterations might occur.^42^ Hepatocytes degenerate, liver sinusoids are hyperaemic with micro-thrombi and can lead to a metabolic disorder. So far, these complications are reported only in severe forms, but less is known about athletes infected with milder symptoms and the potential detrimental effect on liver and kidney of an early return to sport, which would result in misadapted physiological responses to exercise. We then suggest adding specific blood and urine biomarkers to the PPE to help understand the severity of the infection in athletes before a return to sport. Recent work reported that C reactive protein (CRP) levels are increased in patients with COVID-19, and it has been shown that median CRP values are strongly correlated with disease severity and prognosis.^43^ Based on a global review of laboratory markers, complete blood count, interleukin 6, alanine aminotransferase, aspartate aminotransferase, albumin, CRP, lactate dehydrogenase, procalcitonin, ferrintin,^44^ troponin I, CK-MB and NT-pro BNP can be relevant to explore the level of severity of COVID-19 in patients.^16^ ^25^ ^45^ ^–^ ^49^ To prevent asymptomatic acute kidney injury that may result from COVID-19 infection, we recommend assessment of traditional urine biomarkers such as the glomerular filtration rate of the kidney, creatinine, albumin urea, cystatin C, beta-trace protein, beta-2 microglobulin and fatty acid-binding proteins.^50 51^ For a better understanding, biomarkers are presented in table 1. During the first month of practice, tracking the evolution of kidney and liver biomarkers could be of great help to understand the consequences of the viral infection and prevent a putative worsening due to a too rapid and/or too high energy expenditure. In that perspective, oxygen consumption (VO_2_) evaluation could be an additional valuable approach.

**Table 1:** Biomarker assessment recommendation for athletes infected with COVID-19

Haematological parameters	Organ	References
Interleukin 6	Inflammatory	^13 14^
Complete base compte	Inflammatory	^13 14^
Albumin	Liver	^13 14 36^
Alanine aminotransferase	Liver	^13 14^
Aspartate aminotransferase	Liver	^13 14^
C reactive protein	Inflammatory	^13 14 16 19 24 36 39^
Serum creatinine	Kidney	^13 14^
Lactate dehydrogenase	Liver	^16 19 24^
Procalcitonin	Liver	^48^
Ferrintin	Liver	^45^
Troponin I	Heart	^48^
Creatine kinase-MB	Heart	^48^
N-terminal pro b-type natriuretic peptide	Heart	^41 48^
**Urine markers**	**Organ**	**References**
Glomerular filtration rate	Kidney	^51 52^
Creatinine	Kidney	^51 52^
Albumine urea	Kidney	^51 52^
Cystatin C	Kidney	^51^
Beta-trace protein	Kidney	^51^
Beta-2 microglobulin	Kidney	^51^
Fatty acid-binding proteins	Kidney	^51^

## VO_2_ EVALUATION FOR ATHLETES INFECTED WITH RESPIRATORY SYNDROME

The data obtained from various groups worldwide and the 31 provinces of China suggest that the clinical symptoms of COVID-19 are more or less similar to that of SARS-CoV infection.^52 53^

SARS is associated with pulmonary complications in the form of pulmonary fibrosis and bronchiectasis.^54^ A reduction in aerobic exercise capacity 3 months after hospital discharge has been reported in some adult survivors of the SARS-CoV.^55^ This reduction could last up to 12 months after the onset of the illness.^56^ Such impairment was consistently shown to be at variance with the lung function of these subjects, which was either normal or showed only mild abnormalities. Yu *et al* ^57^ reported that aerobic capacity, reflected by peak VO_2_, was also reduced in a cohort of asymptomatic children 6 and 15 months after the acute episode of SARS-CoV. Moreover, whatever the severity of symptoms experienced, because of lock-down, athletes will certainly have a reduction in maximal and submaximal aerobic performance. These losses in aerobic performance, due to detraining, due to sedentarism-induced detraining,^58^ alter cardiovascular function and muscle metabolic potential.^12^ VO_2_ testing is an important clinical assessment tool since it provides a composite evaluation of the respiratory, cardiac and metabolic systems. Exercise testing is thus a more reliable assessment method of functional outcome than resting pulmonary function tests.^44^ In the case of athletes infected with COVID-19 with minor or no symptom and a normal cardiological assessment, a VO_2max_ test must be performed. In more severe cases, a follow-up of the peak VO_2_ and ventilatory threshold as indicator of improving aerobic capacity could be useful, as recommended in patients infected with SARS-CoV.^54 55^

COVID-19 could also have an impact on metabolic adaptation during exercise.^57^ The evaluation of the respiratory exchange ratio could help to understand the effect of the disease on metabolic adaptations to exercise in athletes with moderate symptoms or asymptomatic. In the case of athletes who recovered from a severe infection, complementary measurement should be proposed. Lipid profile assessment before and after exercise and glycaemia measurements during the incremental VO_2_ test are currently performed on patients suffering from metabolic disorder to exercise.^59^ This approach could be proposed to get insights into the consequences of the viral infection on metabolic adaptations to exercise, together with an evaluation of other performance determinants.

## ATHLETES INFECTED WITH COVID-19, LOCK-DOWN AND PHYSICAL PERFORMANCE DETERMINANT

Athletes are used to reduced activity periods throughout their sport careers, usually coinciding with the end of their competition period, illness, injury or other factors. Nevertheless, infected athletes are facing the cumulative effects of the illness and detraining on their physical performance. Because COVID-19 is a new type of coronavirus (SARS-CoV-2) that shares 96% of his genotype with the SARS-CoV of 2002,^5^ one can speculate that they share the same effect on muscles. So and colleagues^60^ reported loss of muscle mass in patients infected with mild and severe form of SARS-CoV. They concluded that it can affect force production and locomotion. Ong *et al* ^61^ suggested that the reduced exercise capacity was probably related to myalgia. Additionally, the reduction in physical activity due to lock-down policies may also have affected muscle mass, and more generally, body composition. We then suggest that the recovery of muscle mass with training may be longer in athletes previously infected with COVID-19. To improve return to practice and follow the effect of training on muscle mass, periodic evaluation of body composition and force production capacity is recommended.

Patients infected with COVID-19 also present central nervous system alterations.^26^ Excepted for asymptomatic, dysgeusia, described as an highly frequent early or lone symptom of COVID-19, and hyposmia or anosmia have been reported, even by professional (basketball) athletes with mild symptoms.^16^ However, the neuro-invasive potential of COVID-19 remains poorly understood, and the impact on neuromuscular function warrants further investigation.^27^ From a practical point of view, it may specifically affect power and force production through modulation of the neural drive.^62^ The countermovement jump (CMJ) is one of the most popular tests to monitor an athlete’s muscle power of the lower extremities. An athlete’s CMJ performance is relevant in a variety of sports and commonly quantified by jump height or flight time, which have been considered as indicators of vertical jump performance^63 64^ and used to measure training adaptations.^65^ The standardised drop vertical jump (DJ) from 30 cm height as a screening tool for evaluation of deceleration activities, eccentric strength, has also largely been reported in the literature.^66 67^ To the best of our knowledge, there is no information about the impact of COVID-19 infection on neuromuscular performance in the literature. Given its potential effects of the central nervous system, evaluating muscle strength and power using simple and classical field-tests could be clinically relevant as a first intention. To get further insights into the effects of COVID-19 on the motor drive, electromyography, twitch interpolation and evoked potentials may be implemented in athletes who have a history of severe COVID-19 infection and/or demonstrate a slow recovery of force/power production capacity.

Moreover, extra attention should be given to the possible post-viral fatigue syndrome,^59^ which may also generate neuromuscular fatigue. The use of CMJ and DJ in isolation as a global indicator of muscle power of the lower limb muscles does not inform about the specific effect of neuromuscular fatigue on sports performance.^68^ Neuromuscular fatigue arises not only because of peripheral changes at the level of the muscle but also because the central nervous system fails to drive the motoneurons adequately.^69^ Appropriate screening methods, combining stimulation methods, dynamometry and electromyography may be used to identify and evaluate the relative contributions of central and peripheral factors to peripheral fatigue.^68^ That could help to prescribe exercise modalities adapted to the level of fatigue and propose countermeasures, such as nutritional and/or specific training regimens, to help athlete to regain performance faster.^70^

**Figure 1:**
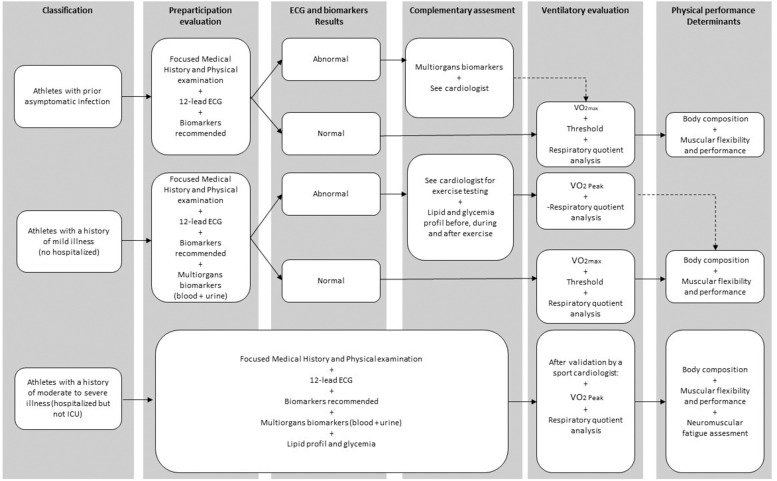
Guidelines for athletes infected with COVID-19 before returning to sport. ICU, intensive care unit; VO_2_, oxygen consumption.

Finally, the fatigue reported by patients with COVID-19 may have affected their flexibility. Indeed, fatigue leads to inactivity, which in turn may decrease flexibility.^71^ Inactivity affects different muscles and muscle chains depending on whether they are tonic or phasic, causing muscle shortening and/or hypertonia or laxity and/or hypotonia depending on the muscle type. The ‘Y Balance Test’ is an easy and reproducible method to assess the flexibility of hip and leg muscles.^72^ Including flexibility evaluation in the PPE would give insight into the flexibility status of athletes with COVID-19 and may ensure a safe return to activity. Managing the combined consequences of COVID-19 infection and lock-down policies on athletes is represented in figure 1. This combined approach based on the physical performance determinant and medical assessment has a dual interest for the sports medicine community, ensuring a return to practice without risk and advising athletes to accelerate their return to performance.

## CONCLUSION

The COVID-19 infection is characterised by influenza-like symptoms, respiratory troubles, and in some cases, multiorgan failure may occur. These effects may combine with the detrimental effects of detraining induced by the lock-down policies. In the context of sport, this may translate into potential alterations of the cardiorespiratory, metabolic and neuromuscular responses to exercise. The present guidelines aim to propose a framework for an easy and accessible assessment of athletes to ensure a safe return to practice and optimise a quick recovery of performance. However, our knowledge of the effects of COVID-19 infection on physiological responses to exercise is very scarce. Research in this field will have to be carried to document this issue and update the proposed framework.

**What is already known?**

- COVID-19 is currently characterised by influenza-like symptoms and respiratory troubles; in some cases, multiorgan failure may occur and especially cardiac injury. The main difficulty is that symptoms of COVID-19 vary among individuals, and athletes may be affected by no apparent sign of the disease. This could be a real danger for athletes when returning to their usual training and play.
- Isolation could have a significant impact on the physical and mental state of the athletes leading to detraining. A reduction in aerobic capacities, muscular power and flexibility and a diminution of muscle mass are expected.
- Combined effect of detraining and COVID-19 symptoms may influence the arousal of post-viral fatigue syndrome, thus influencing the capacities of athletes to perform exercise and increasing the risk of injury.

**What are the findings**

- COVID-19 may lead to multiorgan failure, and especially cardiac and vascular injuries. Allowing athletes to return to practice on the sole basis of cardiac assessment may underestimate risks. To have a more global assessment and insights about the severity of the disease, blood- and urine-specific biomarkers are recommended.
- Since COVID-19 is associated with pulmonary complication, athletes could experience a reduction in aerobic exercise capacity. VO_2_ evaluation is thus recommended to help athletes to improve return to training.
- COVID-19 can have a potential effect of the central nervous system and the motor drive. Electromyography, twitch interpolation and evoked potentials may be implemented in athletes who have a history of severe COVID-19 infection and/or demonstrate a slow recovery of force/power production capacity.
